# The Thirty-Third Annual Meeting

**Published:** 1920-07-03

**Authors:** 


### THE ROYAL NATIONAL PENSION FUND FOR NURSES.

### The Thirty-Third Annual Meeting.

The thirty-third Annual General Meeting of the members of the Royal National Pension Fund for Nurses was held at the Royal Society of Arts, John Street, Adelphi, London, on Wednesday, June 23. Sir Everard Hambro, K.C.V.O., the Chairman of the Council, presiding. Among the letters of regret for non-attendance was one from Mr. George King, F.I.A., F.F.A., the Consulting Actuary, who wrote: "I should nevertheless have liked to have attended on this occasion if only to pay my homage to the memory of our late friend, Sir Henry Burdett. My intercourse with him has lasted through many years, and I deeply deplore his loss. When he was starting the Pension Fund he called me in to assist him in the scientific branch of the undertaking, and since then we have been closely united, and have discussed the affairs of the Fund on innumerable occasions. From the very beginning his whole heart was given to the Fund as one of the many ways in which he worked for the welfare of nurses, and his devotion was unfailing. All those connected with the Fund must deplore his loss, but they are only a few of the multitude whom Sir Henry Burdett tried to benefit throughout the whole course of his long life. His unwearying efforts to do good are an example and stimulus to us all."

### A Memorial to Sir Henry Burdett.

**The Secretary (Mr. Louis H. M. Dick)** having read the notice, **Sir Everard Hambro** said: You will all have noticed that since we last met Sir Henry Burdett has passed away, and I think that, seeing he devoted all his energies and all his life to the service of the nurses, we cannot do less than pass a vote of condolence to-day with his family, and express our sincere sympathy with them as well as our appreciation of the work which he did for the nurses. I have often said that this Royal National Pension Fund for Nurses was created by nurses, sustained by nurses, and supported by them, but I do not think that the Fund could ever have been started had it not been for Sir Henry Burdett. He was the first to see that nurses, out of their emoluments, were able to lay by something for their old age. He was the first to see that it was very advisable for all nurses not only to lay by for their old age, but also to acquire the habit of saving, and that that habit would come much more easily if they had to make fixed payments. I have received several suggestions as to how you could best perpetuate his memory. I do not think you could do it better than by pushing forward the Fund which he established and which he had entirely on his heart. It was the work of his life, the work of which he was proudest. I think if you wish to do anything to commemorate him it should be in that direction. With the assistance of your fellow-sisters you should push forward the interest of the Fund so that by all nurses joining it it shall become, in fact as well as in name, the Royal National Pension Fund for Nurses.

### The Habit of Saving.

You will see from the accounts that there have been about 150 more policies taken out this year than there were last year, but I am sorry to say that a great number of nurses have left us. I think it will surprise you to know that the total amount withdrawn from us this year by nurses who have left us is about £86,000. This is a very large sum. There may be various reasons why they have done so. Possibly they have married; if so, I can but congratulate them. Others may have withdrawn because they were anxious to do their bit by assisting the Government with their money. If that was their reason, I think they must have forgotten that the money we have collected from you ever since the war began has been invested in Government bonds, and that the nurses, together, have also been able to give great assistance to the Government by lending a very large amount of American stocks, or selling those securities to the British Government when that Government wanted them very badly. There may be another reason why nurses have resigned. They might have thought that they were getting a higher rate of interest outside, and that is probably true in the abstract. But I am not quite certain that it will turn out true in reality, because you must remember that having to make certain payments regularly is a great incentive to saving. Not having to make those payments probably means that the savings have not been made. Therefore I think that, in the end, they would really have found themselves better off if they had stuck to the Royal National Pension Fund for Nurses.

### Queen Alexandra's Message.

I may tell you that this morning I sent to Her Majesty Queen Alexandra the following telegram: "The Chairman of the Royal National Pension Fund begs, on behalf of the nurses who hold their annual meeting to-day, to offer to Her Majesty Queen Alexandra their humble greeting on this her Rose Day, and the expression of their hope that she may soon be restored to perfect health." Now I have just received from Her Majesty the following answer: "I thank you very much for the telegram you send me to-day, and please express to all present at the meeting my deep appreciation of their kind thoughts for me. Let me assure you of my continued interest in the Royal National Pension Fund for Nurses, of which I am President, and my sincere sympathy in its aims and objects; I cannot conceive any worthier purpose than the welfare of our nurses to whom the country owes so much, and I wish the good work of the Fund prosperity and success.—Alexandra." (Cheers.)

### Great Services to the Nursing Profession.

**The Chairman** then proposed: "That the nurses assembled in their annual meeting wish to express to the family of the late Sir Henry Burdett condolence on the loss they have sustained and assurance that they, one and all, have appreciated his great services to the nursing profession." This was seconded by Miss Skillman, R.R.C., and supported by Miss Clara Fisher. The motion was carried unanimously.

### Ten Years' Progress.

**Sir Thomas Dewey,** Bart. (Deputy-Chairman), who was received with cheers, said: I happened last week to pick up a report that was discussed in this room one Wednesday in June, just ten years ago. I read it and compared it with the present report. I noticed that 1,077 nurses were then drawing annuities at the rate of £26,000 a year; our report to-day states that we are paying £72,000 a year to 2,723 nurses, and remember we are enabled to pay the extra £46,000 in annuities because our invested funds have increased during the ten years from £1,424,000 to £2,161,000. The Royal National Pension Fund has been a great advantage to nurses in the past. Will it continue to be so during the next ten years? You have heard from our Chairman the number of nurses who are leaving the Fund, and that they are apparently using the Fund more as a savings bank than as a provision for old age. I cannot think that they are wise in withdrawing their money unless under very urgent circumstances. No investment can be more secure than a pension in the Royal National Pension Fund for Nurses.

### The Splendid Example of Nurses.

As to the Junius Morgan Benevolent Fund, for very many years I have had the privilege of assisting in its benevolent work. The Benevolent Fund report speaks of the death of Mrs. Burns, the daughter of Mr. Junius Morgan and the splendid head of our Advisory Committee. Mrs. Burns seems to have lived to help nurses. In consequence of the necessarily high price of every article, the nurses' difficulties have greatly increased, and we are now

**Royal National Pension Fund for Nurses**—(*continued*).

distributing grants amounting to more than £2,000 a year, being £500 more than in the pre-war days. We received last year for the Benevolent Fund donations of £500 each from Mr. Johnston and from Mr. J. Pierpont Morgan. Mrs. Burns—she was always a very generous contributor—left us a legacy of £2,000. We are also splendidly helped by the nurses' shilling subscriptions. I trust they will be not only continued, but increased in number. Nurses are certainly not highly paid, but what a grand example they set to other professions. More than £2,000,000 of their own money is invested for their old age, and more than 1,000 nurses annually send subscriptions to the Benevolent Fund to assist their sisters who are in distress.

The report was carried unanimously.

The ballot for the Policyholders' representatives was then announced, Miss Hogg, C.B.E., and Miss Smith, A.R.R.C., having been elected.

Resolutions were then proposed that the retiring members of the Council, Sir Everard Hambro, K.C.V.O., Sir Thomas Dewey, Bart., Sir Eric Hambro, K.B.E., Mr. J. Pierpont Morgan, and the Right Hon. Sir Archibald Williamson, Bart., M.P., being eligible, should be re-elected, and that Messrs. Whinney, Smith and Whinney should be re-elected as auditors, which were carried.

**Mr. Schooling** then proposed a very hearty vote of thanks to Sir Everard Hambro for taking the chair, which was carried by acclamation.

Before separating, Sir Thomas Dewey said he was sure they should all like to express to Mr. Dick their congratulations on his recovery from his recent illness, and their pleasure at having him back with them. He also thanked Mr. Facy, who had charge during Mr. Dick's absence, and the excellent staff in Buckingham Street at the offices of the Pension Fund.

**Mr. Dick** said how much both Mr. Facy and himself appreciated the kind words.

The proceedings then terminated.

